# Populations, Traits, and Their Spatial Structure in Humans

**DOI:** 10.1093/gbe/evab272

**Published:** 2021-12-11

**Authors:** Mashaal Sohail, Alan Izarraras-Gomez, Diego Ortega-Del Vecchyo

**Affiliations:** 1 Department of Human Genetics, University of Chicago, USA; 2 Centro de Ciencias Genómicas (CCG), Universidad Nacional Autónoma de México (UNAM), Cuernavaca, Morelos, México; 3 Laboratorio Internacional de Investigación sobre el Genoma Humano (LIIGH), Universidad Nacional Autónoma de México (UNAM), Juriquilla, Querétaro, México

**Keywords:** complex traits, populations, genetic structure, spatial variation

## Abstract

The spatial distribution of genetic variants is jointly determined by geography, past demographic processes, natural selection, and its interplay with environmental variation. A fraction of these genetic variants are “causal alleles” that affect the manifestation of a complex trait. The effect exerted by these causal alleles on complex traits can be independent or dependent on the environment. Understanding the evolutionary processes that shape the spatial structure of causal alleles is key to comprehend the spatial distribution of complex traits. Natural selection, past population size changes, range expansions, consanguinity, assortative mating, archaic introgression, admixture, and the environment can alter the frequencies, effect sizes, and heterozygosities of causal alleles. This provides a genetic axis along which complex traits can vary. However, complex traits also vary along biogeographical and sociocultural axes which are often correlated with genetic axes in complex ways. The purpose of this review is to consider these genetic and environmental axes in concert and examine the ways they can help us decipher the variation in complex traits that is visible in humans today. This initiative necessarily implies a discussion of populations, traits, the ability to infer and interpret “genetic” components of complex traits, and how these have been impacted by adaptive events. In this review, we provide a history-aware discussion on these topics using both the recent and more distant past of our academic discipline and its relevant contexts.

Significance

Complex traits such as height, educational attainment, or diseases like hypertension vary across the globe due to the interplay of genetics and the environment. In this review, we describe the evolutionary processes that could influence complex trait variation and highlight the challenges of separating the environmental and genetic contribution to a complex trait. We discuss these topics in light of the advantages and limitations of current approaches for studying the genetic basis of complex traits.

## Introduction

One of the main questions of interest in human genetics is to understand the genetic factors driving the phenotypic diversity observed among individuals. This is a difficult enterprise because the majority of the phenotypes of interest are complex traits that are jointly determined by many genetic loci and environmental variables. Genome-wide association studies (GWAS) have allowed us to make progress in identifying the genetic loci that influence the phenotypic diversity of a complex trait in a particular study cohort and environment. This has allowed us to start identifying similarities and differences in the genetic architecture of a complex trait across human diversity. Here, the genetic architecture is defined by the number, effects, frequencies, and heterozygosity of the genetic variants changing the value of the trait in a particular environment, taking into account the interactions of the variants with each other and the environment (Timpson et al. 2018). The unraveling of genetic architectures around the world raises the question of what particular factors could be driving observed differences in traits, how these factors are influenced by the environment, and how the phenotypic diversity observed in humans across the globe are driven by the joint effects of the genetic architecture of a trait and the environment. Here, we emphasize these issues by describing the evolutionary and historical processes driving differences in the global distribution of genetic variants and complex traits. We also discuss the accuracy of current genotype-based approaches to predict phenotypes and detect polygenic adaptation given variable environments and genetic architectures. We begin our discussion with a historical perspective on the definition of a population and a complex trait, and we end our review with a history-aware contemplation to help guide the trajectory of future research.

## Populations in Biology

Researchers typically try to make sense of biological variation by grouping individuals based on shared observed characteristics. This is a complicated task that can be approached differently depending on the purpose of the definition that we seek. Focusing on natural populations, Waples and Gaggioti (2006) categorize two main approaches to define populations, the ecological approach and the evolutionary approach. The first one proposes that individuals of a species belong to the same population based on interactions within the same space and time but not necessarily requiring reproduction, whereas the latter requires the potential for reproduction which implies being in close enough proximity to mate (Waples and Gaggiotti 2006). However, the delimitation of a population is complicated due to the distribution and migration of individuals through space. For instance, if two populations are interconnected through constant gene flow overtime, should they be considered separate populations or at what point should they be considered one population? Another related complication is the definition of the space that they inhabit; how should the area of a population be delimited, if it all? Moreover, what happens if the ranges of two populations overlap? If they share a fraction of their range, then are they two separate populations or part of a single more extensive range?

Despite the problems of the definition of populations in space, the concise definition of populations in theoretical models has been useful to advance our understanding of the spatial patterns of genetic diversity. As an example, models of populations with predefined sizes and migration rates that have a clearly delimited space have helped us elucidate important concepts such as the patterns of isolation by distance (IBD). IBD is a very robust prediction derived from a mathematical framework and posits that there will be more genetic differences between individuals that are farther away from each other geographically under a continuous spatial range (Wright 1943). This overall general pattern has been observed in humans (Relethford 2004; Ramachandran et al. 2005). Even though IBD is a good model for the genetic dynamics of populations, the pattern can be disrupted by the simple fact that there are areas harder to get to than others but also because of humans’ unique capability of long-distance group migration. For example, when looking at present-day cosmopolitan societies, we would find IBD disruption because of the historical large-scale migration events from a variety of distinct geographical locations. We note that this is not an exclusively contemporary phenomenon and has happened throughout history (e.g. Lazaridis et al. 2014). Perhaps an aid to understanding the picture of human variation would be to incorporate both dimensions of time and space to current population models (Bradburd and Ralph 2019). Human variation can be neither thought of as many small isolated populations nor as one large global population, because boundaries are blurry in space and time.

**Fig. 1.**
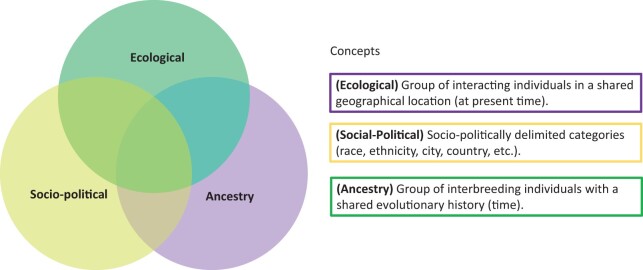
Ambiguity between different population concepts. We illustrate three different population concepts: The ecological definition in purple, the sociopolitical definition in yellow, and the ancestry-related definition in green. Even though these three concepts may have some overlap, they allude to different processes.

It is important to note here that idealized population definitions that aid the development of mathematical models do not generally present an accurate representation of present-day human diversity, hence one should be wary when mapping these constructs to empirical biological data. In practice, the fields of human genetics and genomics have grouped individuals into populations with a certain ambiguity, where populations have been used to refer to races, ethnic groups, individuals sharing genetic ancestry (see Appendix—Keywords), a nationality, a religion, or a geographic region, with multiple definitions used interchangeably (Panofsky and Bliss 2017). Although some of these concepts may overlap, referring to them ambiguously under the population label can also lead to misunderstandings (fig. 1). Continental ancestry labels have emerged as the most common way to group in recent decades (Panofsky and Bliss 2017). However, their use has been critiqued due to their conflation with racial categories, ambiguity in how to define ancestry, and their use artificially delimiting a continuous variation space, as well as highlighting only a specific time slice of the true historical notion of ancestry (Lewis et al. 2021).

To prevent such essentialization of discrete ancestry labels, it is reasonable to describe them as outputs of specific methods such as admixture with certain assumptions, and label them as ancestry from present-day Europe instead of European ancestry for example. Furthermore, when determining how to analyze individuals in genetic and genomic studies, it would be advisable to select grouping criteria that are most in alignment with the research questions being asked, as well as clearly stating such criteria and why they are important for the analysis. For example, how do you define and label groups and why? Should your grouping be defined by genetic ancestry (and at which time slice), self-identified race, or some other variable? As such, it is also advisable to group only when it is justified by the research question and not by default. Additionally, when sampling is limited or not uniform across a grouping criterion, it should be pointed out that there is insufficient data to generalize particular results to the larger group.

## Traits in Biology

The concept of population is commonly used to group individuals for further analyses including those of trait variation. Traits are often described as a phenotypic characteristic that can be either measured or directly observed in an organism. One particularly useful definition was put forward by Dobzhansky who defined a “visible trait” as “the outcome of certain developmental, physiological and ultimately physico-chemical processes in the organism” (Dobzhansky 1956) noting that these processes come from underlying genetics but are also influenced by the environment. This definition implies that traits are the conjunction of distinct processes and thus can be conceptualized as a group of characteristics that might be made up of another grouping at different physical scales. For example, one of the most discussed traits in humans is height, but height is just an arbitrary observation that can be broken down into different components such as “femur length” trait or a “growth hormone production” trait that can be thought of as different traits. A trait is not only a conjunction of distinct biological processes, but it is a conjunction of these and other environmental processes, as well as their interactions. For example, height is also affected by nutrition, because nutrition may compensate for metabolite deficiencies as well as change the expression of the genes affecting height (Perkins et al. 2016).

Traits such as height that show continuous variation are referred to as “quantitative” or “complex” traits. Common disorders that seem binary such as Type 2 Diabetes (T2D) are also quantitative traits (Plomin et al. 2009). R.A, Fisher famously demonstrated that the observation of continuous variation can be explained by the contribution of many different genetic Mendelian factors to changes in the value of a phenotype (Fisher 1918). However, it must be noted that a purely environmentally determined trait can be continuously distributed as well. R.A. Fisher also showed that the larger the number of loci involved in a trait, the lesser the individual contribution of each locus to the trait. The fact that many mutations can impact the expression of a phenotype implies that the genetic background is of importance to the trait. The genetic background can manifest in the expression of a trait through additive effects, epistatic interactions among genetic variants, and through pleiotropic effects of genetic variants that affect many traits simultaneously.

**Fig. 2.**
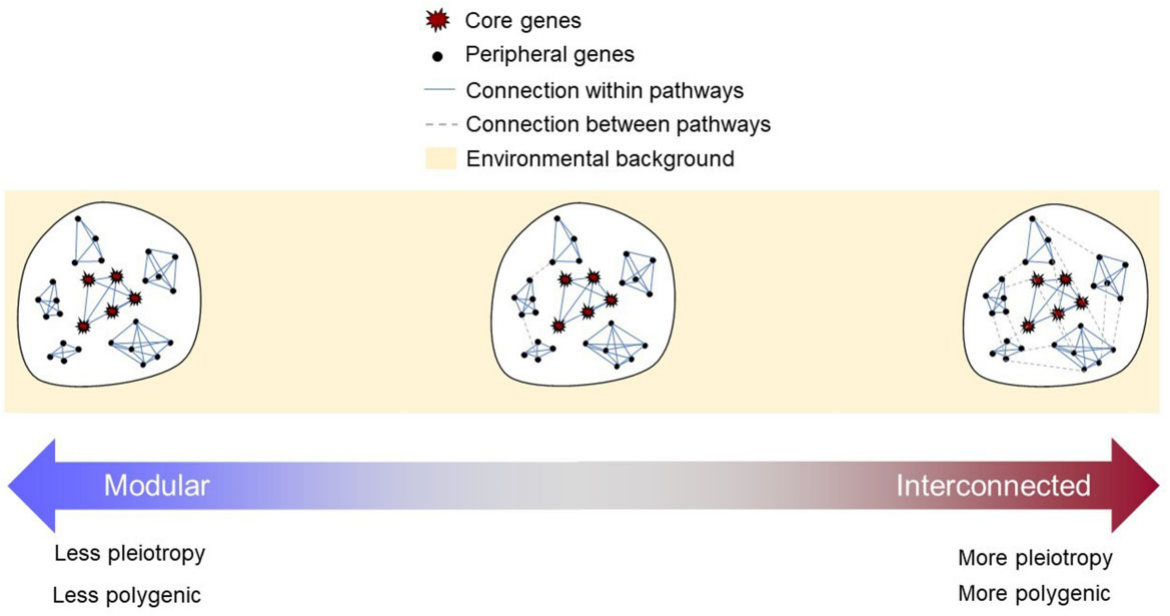
Genetic background, pleiotropy, and polygenic trait architecture. Polygenic traits have core genes that have a high contribution to the trait because they are directly related to the main biological pathways that produce the trait. Polygenic traits also have a contribution from peripheral genes, which are not directly related to the core pathway, but because they may be connected to core genes they still exert an effect. If the genome behaves modularly, meaning that pathways are grouped into discrete clusters with little or no communication between each other, then the trait is only primarily influenced by changes in core genes. On the other hand, if the genome is highly or completely interconnected (as the omnigenic model suggests; Boyle et al. 2017), then peripheral genes have a bigger impact than core genes. This phenomenon is due to the peripheral genes significantly outnumbering the core genes. Even if the peripheral genes have very small effects, they will have a greater effect on the trait than core genes because of their larger number. According to this view, the more modularly the genome behaves, the less pleiotropic and polygenic a trait will be. On the other hand, with a more interconnected genome, we could expect traits to be more pleiotropic and polygenic. As illustrated by the environmental background in yellow, we assume no environmental variability in this figure.

Finding statistically conclusive support for epistasis is a challenging task mainly due to it having small effects, and its contribution to complex traits remains to be accurately quantified (Wei et al. 2014). On the other hand, pleiotropy is an important phenomenon driving the evolution of complex traits. A study looking for signatures of pleiotropy used GWAS data, which we will explain in the next section, from 558 unique traits and found 41,553 trait-associated loci across the analyzed traits (Watanabe et al. 2019). Those traits were classified into domains that share a particular function such as a neurological domain or metabolic domain. Then, the authors defined physical blocks in linkage disequilibrium (LD) with the trait-associated loci and found that 93.3% of these blocks were associated with more than one trait, whereas 90% were associated with more than one functional domain. These results could be explained by genes or single nucleotide polymorphisms (SNPs) having a pleiotropic effect or, alternatively, by having two genes or SNPs being in close enough proximity such that they are part of the same LD physical block. Further analysis made by the authors found that 67.2% and 32.4% of the genes and SNPs associated with a trait had an impact on more than one functional domain, respectively. These results show that pleiotropy at the SNP and gene level is an important phenomenon acting on multiple traits with different functions (Watanabe et al. 2019). The presence of pleiotropy and the possible effects of epistasis in complex trait variation highlights the importance of the genetic background. Additionally, the evidence that pleiotropy seems to be widespread tells us something about the architecture of complex traits; that biological pathways leading to the expression of a phenotype are not isolated from the biological pathways of other phenotypes. If the majority of these pathways are interconnected, then we would expect genetic variants changing the enzymatic activity of those pathways to be more pleiotropic and traits to be more polygenic (fig. 2). Another consideration is that because traits are arbitrary in terms of how they are measured or defined, the definition itself may account for the trait having a different degree of pleiotropy. In this sense, we could assume that a more “broadly” defined trait, such as body mass index (BMI) which combines weight and height, could have higher pleiotropy than a more “narrowly” defined trait such as growth hormone production.

**Fig. 3.**
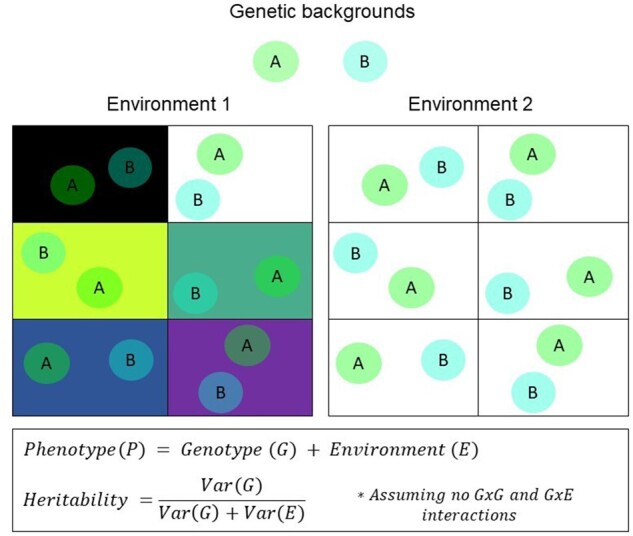
Genes, traits, and their environment. Genes are expressed in a particular environment. Shown here is a visual representation of the expression of a color trait from different genetic backgrounds (*A* and *B*) in different environmental conditions (Environment 1 and 2). Here, a color trait is simply the sum or superposition of the genetic background color (shown by the circles at the top of the figure) and the environmental color (shown in each tile), that is P = G + E. Environment 1 has a high variance, demonstrated by different conditions or background colors, whereas Environment 2 has a low variance demonstrated by a single background color. Genes under different conditions (Environment 1) result in color traits that are somewhat different and differences in color are both influenced by the environment and the genetic background. Notice how in some sections of Environment 1, the two genetic backgrounds are distinguishable, whereas in others, the two genetic backgrounds are very much alike (e.g. white background vs yellow background). In Environment 2, however, there is only one predominant condition of the environment which makes the genetic background a bigger determinant of the color trait instead of the environment. The measured heritability of the trait from Environment 1 would be lower compared with the heritability measured in Environment 2 simply because the latter environment is more homogeneous.

Beyond genetic background, complex traits can be heavily influenced by the contribution of the environmental background that alters the processes in the organism giving rise to the trait. These gene by environment interactions and environmental effects make it complicated to assess how much of the variation of a trait comes from genetics or the environment. In this sense, estimating heritability is always a local analysis (Feldman and Lewontin 1975), for either narrow or broad-sense heritability. It can tell us the genetic contribution to phenotypic variation for a given trait in a particular environmental background, but the implications of those results are challenging to generalize to individuals living in another environment. More broadly, genetic effects must be understood in the environmental conditions under which the genes are expressed (Feldman and Lewontin 1975). Therefore, heritability is not a property of a trait, but rather of the distribution of a trait in a given environment. Furthermore, these heritability estimates can contain masked effects of environmental factors that are not variable in the cohort used to estimate heritability. Heritability is highest when relevant environmental inputs are uniform across a sample, and shrinks as environmental input becomes more varied (fig. 3). A high heritability score does not necessarily tell us whether a trait is primarily genetic; high heritability can also be an indicator of environmental homogeneity (Uchiyama et al. 2021).

## Estimating Genetic Effects and Predicting Complex Traits

### GWAS Overview

**Fig. 4.**
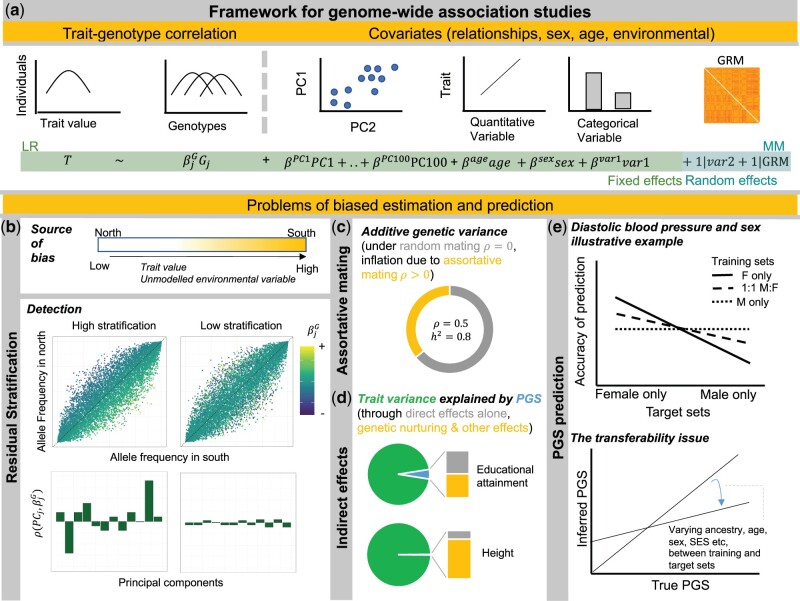
Estimating genetic effects and predicting complex traits. (*a*) Framework for genome-wide association studies. Genotypic values are correlated with trait values in genome-wide association studies using linear regression analysis or mixed-models. Linear regression (LR) analyses only model fixed effects, whereas mixed models (MM) can also model random effects. Principal components are generally included in the model as a fixed effect, and/or the genetic relationship matrix (GRM) is included as a random effect to correct for population stratification. Factors such as age, sex, socioeconomic status, and other environmental variables may be included as a fixed or random effect as well if they are correlated with the trait of interest in the study cohort. Here, the trait distribution (T) in the study cohort is modeled using a set of predictors—genotype at each genetic locus ($G_{j}$), principal components 1–100 (PC1-PC100), age, sex, a variable such as socioeconomic status (var1) as a fixed effect, and variables such as city (var2) and the genetic relationship matrix (GRM) as random effects. (*b*) Residual stratification. If an environmental gradient varies along the same axis as the trait value, and is not appropriately controlled for using PCA or inclusion of said variable in the model, any SNP that is differentiated in allele frequency along the same axis will have an artificially inflated genetic effect size estimated using GWAS. The resulting residual stratification has been detected using two main approaches (Sohail et al. 2019). (*top*) Visualizing estimated genetic effect sizes ($\beta_{j}^{G}$) along the axes of allele frequencies computed within groups reflecting the axis of variation. If $\beta_{j}^{G}$ beta is systematically estimated as positive among alleles with a higher frequency in the south compared with the north in this illustrative example, this likely reflects residual stratification (*left*). Under a case of low stratification, effect sizes would be more evenly distributed (*right*). Correlation (ρ) between $\beta_{j}^{G}$ and the PC SNP loadings (${PC}_{j}$) for each SNP for principal components 1–12 (*bottom*). If the estimated genetic effects are significantly correlated with SNP loadings along a PC that reflects geographic and other environmental structure in the study cohort, that is due to residual stratification (*left)*. In the case of low stratification, effect sizes will not show strong correlations with SNP loadings along any PC (*right*). In general, such correlations can also result from polygenic selection, but the resulting correlation is likely much smaller than those induced by residual stratification, and warrants further study. (*c*) Assortative mating. Under assortative mating (phenotypic correlation between mates ρ>0), the additive genetic variance at equilibrium is inflated compared with under random mating. Here, shown for ρ=0.5, and heritability ($h^{2}$) = 0.8 using equations from (Yengo and Visscher 2018). (*d*) Indirect effects. Using results from (Kong et al. 2018) for two illustrative traits in the Icelandic (deCODE) data, we show the proportion of the trait variance accounted for by the polygenic score (*blue*), and the portion of it accounted for by the direct genetic effect alone (*gray*), or by the genetic nurturing and other indirect indirect effects (*yellow*). The trait here is adjusted by sex, year of birth, and 100 PCs, and the proportion of variance explained by the polygenic score for the unadjusted trait would be lower (Kong et al. 2018). (*e*) Polygenic score (PGS) prediction. Using results from (Mostafavi et al. 2020), we present an illustrative example for diastolic blood pressure (DBP) (*top*). The accuracy of prediction using the PGS for DBP depends both on the gender breakdown of the training sets used to estimate effect sizes and the gender breakdown of the target sets in which prediction is done. The correlation between inferred PGS and true PGS depends on many factors that can vary between the training and target sets (*bottom*). These include genetic ancestry, age, gender, socioeconomic status (SES), etc. Prediction can be further complicated by varying strengths of selection along the genome (Shi et al. 2020).

GWAS are our current best tool to analyze the genetic basis of complex traits in humans (Young et al. 2019). To do this, GWAS estimates the effect of SNPs in the genome with respect to the odds of having a disease, or a certain value of a quantitative trait. Trait values and genotypes are measured in a study group. Trait values and genotypic values are then correlated in a statistical model. If allele frequencies for a SNP are differentiated among individuals with the disease compared with those without the disease, a significant effect size for the SNP is estimated (fig. 4). Because other factors in the study group can cause allele frequencies to differentiate in the same direction as trait values, the model generally includes variables for gender, age, and estimates of population structure to control for their confounding effects in the estimation of genetic effect sizes. Indirect genetic effects and assortative mating may also confound estimation of effect sizes. Furthermore, the study group is a sample with a specific distribution of age, gender, socioeconomic status (SES), geographic location, other environmental factors, and genetic ancestry. Therefore, it is important to consider how well the estimated SNP effects for a complex trait are meaningful in a different study group. We will first overview the approaches currently used to estimate genetic effect sizes and correct for confounding variables, discuss how genetic effects can be overestimated due to insufficient modeling of confounding variables, and end by considering problems of transferability beyond the study group (fig. 4).

### Approaches

Two primary statistical models are used to estimate genetic effect sizes (fig. 4*a*). The first is a linear regression model. This model generally uses principal components computed on the study group to correct for population structure (Price et al. 2006). The principal components capture allele frequency differentiation along major axes of genetic variation. Principal components are normally computed on common variants and may not appropriately control for recent fine-scale structure (Mathieson and McVean 2012). A common approach has been to meta-analyze the effect sizes estimated from a number of smaller studies to boost power. The second model used to estimate genetic effect sizes is a mixed model approach. In this model, a genetic relationship matrix is computed on the study group, and is used to correct for population structure (Loh et al. 2018). Sibling-based GWAS is an alternative approach used to estimate genetic effect sizes, which can circumvent issues of confounders discussed below. However, this approach suffers from low power, due to the paucity of phenotypic and genetic data collected on related individuals, and can bring other confounding issues (Fletcher et al. 2021).

### Problems of Biased Estimation of Genetic Effects

#### Due to Trait-Relevant Environmental Structure

Genetic effects can be overestimated if an unknown environmental factor is differentiated along the same axis as the studied trait (Berg et al. 2019; Sohail et al. 2019). This can happen, for example, if an environmental factor (such as diet) that affects a trait of interest (such as height) varies along the same axis as the trait (fig. 4*b*). If the factor is not appropriately controlled for, the genetic effect partly serves as a proxy for this environmental effect on the trait and, is therefore, overestimated. This phenomenon, often called residual stratification, remains an unsolved problem in complex trait genetics studies (Berg et al. 2019; Haworth et al. 2019; Kerminen et al. 2019; Sohail et al. 2019; Refoyo-Martínez et al. 2021). Two methods used to detect residual stratification (Berg et al. 2019; Sohail et al. 2019; Refoyo-Martínez et al. 2021) are presented in figure 4*b*. These and similar approaches have been recently used to show that residual stratification particularly remains a problem for large meta-analyses of association studies (Berg et al. 2019; Sohail et al. 2019; Refoyo-Martínez et al. 2021). One reason may be that the individual studies used in the meta-analysis are small and therefore cannot appropriately control for population structure using principal components. However, residual stratification concerns have also been found in more homogenous studies such as the UK Biobank (Haworth et al. 2019; Kerminen et al. 2019). This may be because the standard approach of computing principal components on common variants do not appropriately correct for recent population structure (Zaidi and Mathieson 2020), and may be improved by using principal components computed from rare variants or identity-by-descent segments (Byrne et al. 2020; Zaidi and Mathieson 2020). Although residual stratification remains in genetic association studies, the genetic effect assigned to a trait will be inflated and not correctly assigned to an environmental or partly environmental factor.

#### Due to Assortative Mating and Indirect Genetic Effects

Correlation among causal variants induced by assortative mating can confound their genetic effect estimates from GWAS, inflating the additive genetic variance beyond its value under random mating (Yengo and Visscher 2018; Kim et al. 2020; Young et al. 2020) (fig. 4*c*). Genetic effects can also be overestimated due to indirect genetic effects (Kong et al. 2018). We define direct genetic effects as changes in the phenotype in one individual due to its genetic variants. On the other hand, indirect genetic effects result from genetic variants in one individual that have an effect on the trait of another individual through changes in the environment. A form of indirect genetic effects is genetic nurture, where the genetic variants present in parents and other relatives can change the trait of an individual (Kong et al. 2018). Using data of individuals from Iceland with at least one parent genotyped, Kong et al. estimated the trait variance for EA and height among other traits explained by their polygenic score (PGS), which captures the direct effect, genetic nurturing, and other effects such as assortative mating. The PGS is computed by summing the alleles at all trait-associated loci carried by an individual or population, weighted by their “effect size” on a trait as estimated in a GWAS. The score is a genetic predictor of a given trait in an individual or group, and can be understood to reflect the predisposition for that trait based on genetics. The authors use data from the PGS of the alleles not transmitted from parents to their children to compute the trait variance explained by only the direct effect of the PGS. The authors estimate the direct effect of the PGS by computing the difference between the estimated effect of the PGS in the alleles transmitted from parents to children minus the estimated effect of the alleles not transmitted from parents to children. This calculation cancels the impact of genetic nurturing, assortative mating, and other effects in the PGS. In the case of EA, 4.98% of the trait variance is explained by the transmitted PGS, whereas only 2.45% of the trait variance can be attributed to direct genetic effects of the PGS pointing to the importance of indirect effects, such as genetic nurturing, as well as other confounding effects such as assortative mating on the inflation of the trait variance explained by the PGS (fig. 4*d*).

### Problems of Phenotypic Prediction

#### Across Ancestry

Investigating the relationship between spatial genetic and trait structure often requires performing predictions of complex phenotypes using SNP effects recovered in GWAS (fig. 4*e*). These predictions are performed using PGSs (Torkamani et al. 2018). The final trait value is mediated by both the genetic predisposition and the environment in a given individual or group. In the simplest scenario, with no interactions between genetics and the environment, the trait will be the linear sum of the genetic predisposition (PGS) and the environment. There are several potential issues with the estimation of effect sizes (and thus PGS), and their transferability from the study cohort to a new cohort, depending on the specifics of the study cohort and the design of the GWAS. The main factors that contribute to the accuracy of these predictions are past demographic history, natural selection, environmental effects, and genotype by environment interactions.

Past demographic history plays an important role in the prediction of phenotypes. Its impact is mainly due to a larger genetic divergence (as measured by Fst) between the training population used to obtain the effects of associated SNPs via a GWAS study and the target population where we perform the predictions. This impact is manifested in two main ways: differences in the frequency of causal alleles and varying patterns of LD (Rosenberg et al. 2019). Allele frequency differences can lead to causal alleles that are present only in a single population, also known as population-specific causal alleles. The condition of a causal allele being population-specific is dependent on the sample sizes from the included populations in the study. If we include the genetic information from more individuals that are part of other designated populations, then the allele could be found in other populations and lose its condition of being population-specific. Taking this caveat into account, population-specific causal alleles decrease the accuracy of the phenotypic predictions in the target population, because they are not present in the training population where we obtained the SNP effects (Durvasula and Lohmueller 2019). The differences in allele frequencies between populations also contributes to misestimations of the allele effects, because associations with causal alleles that are at low frequency in the target population are harder to detect (Kim et al. 2018). LD between the tagging and causal alleles varies between different populations due to the joint effects of recombination and specific demographic histories contributing to the misestimation of allele effects (Martin et al. 2017). These two manifestations of past population demographic history are likely important for the accuracy of the computed PGSs, although their relative importance remains unclear.

Potential variation of SNP effect sizes in different human populations may be another factor affecting the accuracy of PGSs. A recent study examined this problem by looking at the cross-population genetic correlation between effect sizes in regions of the genome with a particular functional annotation (Shi et al. 2020). The authors found a lower cross-population genetic correlation in regions of the genome that are under stronger background selection. The strength of background selection is correlated with Fst values in human populations (Torres et al. 2018). This correlation suggests that the differences in effect sizes between populations in regions of stronger background selection could be due to allele frequency differences driven by higher drift in these regions without the need to invoke shifting natural selective pressures acting directly on causal alleles. Additionally, Shi et al. 2020 finds that functionally relevant regions of the genome, such as super enhancers, promoters, and regions conserved in mammals, also show a depletion of the cross-population genetic correlation. The authors conclude that including loci from regions that affect gene regulation, are conserved, or are in a region of strong background selection is not the best strategy to perform cross-population phenotypic predictions, because the effect sizes on those regions do not possess a high cross-population genetic correlation.

Furthermore, as will be seen in the “Genetics and Environment Shape Complex Trait Distributions” section, prediction of a certain trait can be poor across study groups simply if the environment is different across study groups because the trait is the sum of genetic and environmental effects. Finally, the presence and importance of genotype by environment interactions in complex trait prediction can lead to poor phenotypic prediction and warrants further theoretical and empirical studies. As an example, the importance of genotype by environment interactions has already been demonstrated for BMI using variants from the *FTO* locus (Young et al. 2016).

Simulations under a simple demographic model that includes African, European, and Asian populations have shown that larger genetic divergences between the target and training population lead to more inaccurate predictions using PGS (Martin et al. 2017; Ragsdale et al. 2020). These simulations were done assuming a genetic architecture without G × G or G × E interactions, where natural selection has no impact on the trait, and where the effect of an allele is sampled from a normal distribution whose variance is dependent on the number of causal alleles and the heritability of the trait (Martin et al. 2017; Ragsdale et al. 2020). Different assumptions about the genetic architecture of the trait could impact the negative correlation of the genetic divergence between populations and the prediction accuracy of the PGS. Changes in the genetic architecture of a trait should be taken into account when analyzing different traits across human diversity.

Moreover, a recent study showed through extensive simulations that the relative accuracy of PGS based on genome-wide significant SNPs can be predicted accurately from modeling LD, minor allele frequencies, and cross-population correlations of causal SNP effects and heritability (Wang et al. 2020). Through theoretical and empirical quantification, they found that LD and allele frequency differences between ancestries can explain between 70% and 80% of the loss in relative accuracy of European-based PGSs in African ancestry for traits like BMI and T2D. These results suggest that causal variants underlying common genetic variation identified in European ancestry GWAS are shared across continents for some traits. However, this remains an area of active research and further theoretical and empirical studies are needed on more cohorts across the globe to define what are the most important factors that impact the accuracy of PGS. As we describe next, factors such as age, sex, and SES can be part of the explanation for observations of variable phenotypic prediction accuracy across ancestries.

#### Within Ancestry

Not only should one be careful when trying to extrapolate phenotypic predictions from genotype data of people who are distantly genetically related, but care should also be taken when doing so even for individuals with a more shared genetic ancestry. A recent study examined the portability of PGSs in individuals with shared “White British” ancestry but differing group characteristics for three exemplary traits: diastolic blood pressure, BMI, and years of schooling (Mostafavi et al. 2020). The values of these traits are correlated to known environmental factors. In the case of diastolic blood pressure, the strongest nongenetic predictor was sex, for BMI, it was age, while for years of schooling, it was SES. For diastolic blood pressure, they constructed two prediction cohorts composed only of randomly selected males or females each while conducting a GWAS with a cohort of equal sex ratio (fig. 4*e*). When the PGS using the GWAS was applied to the female-only prediction cohort, this had a 1.15-fold higher prediction accuracy than when applied to the male-only prediction cohort. Similarly, for BMI and years of schooling, the prediction accuracy was variable when applied to stratified prediction cohorts. In BMI, it was 1.4-fold higher in the youngest group compared with the oldest group and in years of schooling, PGS was shown to be 2-fold more predictive when applied to the lowest SES group compared with the highest one.

To investigate this further, the authors then constructed stratified GWAS cohorts for each trait. A diverse set with an equal ratio of the grouping characteristics and two additional sets made from individuals with a selected group characteristic. For blood pressure, the PGS resulting from the female GWAS set was more predictive (1.35-fold) in a female prediction set. In turn, the PGS resulting from the male GWAS cohort was similarly accurate for both sexes. Similarly, BMI and years of schooling were also sensitive to the choice of GWAS cohort. When PGS was constructed from GWAS of the youngest group (BMI) and lowest SES (years of schooling), it had a higher prediction across all groups. Whether these effects are widespread across other traits or group classifications is unknown, but for these three traits, prediction accuracy of PGS can vary in individuals of a shared genetic ancestry based on sex, age, and SES. A broader conclusion from these results is that the prediction accuracy of PGS between individuals with a different ancestry will also be affected by factors, such as SES or age, when those factors change between groups.

### Potential Solutions to Problems of Prediction

Some solutions have been proposed to improve the prediction of phenotypes using data from multiple studies. One potential problem is that phenotypic predictions in a particular target population can be inaccurate because they are based on GWAS data using a small sample size from that target population. Due to this small sample size, the effect sizes estimated are inaccurate and lead to poor phenotypic predictions. However, one solution that has been proposed to tackle this problem is to create a multiethnic PGS that includes a linear combination of two PGSs from: 1) A study cohort where accurate effect sizes can be obtained due to a large sample size available, and 2) a study cohort with less accurate effect sizes due to a smaller sample size available (Márquez-Luna et al. 2017). This approach uses the linear combination of PGSs from two different cohorts to create a multiethnic PGS that increases the accuracy of the phenotypic predictions. This multiethnic PGS can also include information from principal component analysis to adjust for ancestry. Broadly, the multiethnic PGS boosts the phenotypic predictions by leveraging the information from two studies performed in two different cohorts and does not make any explicit assumption about the genetic architecture of the analyzed trait. The method has been tested in simulations that assume shared genetic effects and shows improved performance compared with using a PGS that only uses genetic effects data from a single population (24–260% improvements in prediction accuracy). This approach improves the prediction accuracy of T2D by more than 70% in cohorts of Asian and Latino populations compared with approaches that only use effect size data from a single population (Márquez-Luna et al. 2017). A more recent method, PRS-CSx, relies on a similar idea to combine summary statistics from multiple populations, explicitly assuming that genetic architecture is mostly shared across populations (through a shared continuous shrinkage prior and leveraging LD diversity across discovery samples) but allowing for population-specific effect sizes as well (Ruan et al. 2021). PRS-CSx has been shown to outperform alternative methods for polygenic prediction across a range of genetic architectures and ancestries (Ruan et al. 2021).

A few studies have built up on the approach of using effect sizes from different populations to build better phenotype predictors for admixed individuals by leveraging the local ancestry background of each chromosome (Bitarello and Mathieson 2020; Marnetto et al. 2020). These studies propose to use effect sizes that depend on the ancestry background of each allele to compute a PGS. These methods assume that effect sizes vary across ancestries and, therefore, taking into account those differences should improve phenotypic predictions. The ancestry-specific effect sizes can be obtained from GWAS conducted in cohorts with different ancestries. Alternatively, a recently developed software package called Tractor can be used to estimate ancestry-specific effect sizes by leveraging local ancestry estimates in admixed individuals (Atkinson et al. 2021). These ancestry-specific effect sizes can be used to create a new PGS (Bitarello and Mathieson 2020) that modestly improves height predictions by 0.1–0.3% in African Americans compared with two different constructions of a multiethnic PGS following (Márquez-Luna et al. 2017). The authors suggest that an improvement of these predictions will depend on studies with larger sample sizes in African populations (Bitarello and Mathieson 2020). Consistent with this prediction, another study creates a different PGS also leveraging ancestry-specific effects sizes (Marnetto et al. 2020). Using ancestry-specific effect sizes from a European cohort taken from the UK Biobank, and from an Asian cohort taken from the Biobank Japan, the authors compute a combined ancestry-specific PGS. The study finds modestly better predictions for height in admixed East Asians in terms of the *R*^2^ values compared with PGSs that only use effect sizes from one particular ancestry (Marnetto et al. 2020).

Such local ancestry approaches implicitly assume varying genetic architecture (different effect sizes and allele frequency of causal alleles) by ancestry and have only shown modest improvement in polygenic prediction. Meanwhile, the PRS-CSx approach assumes a mostly shared genetic architecture across ancestries, and boosts power by leveraging summary statistics and LD information across populations while allowing for population-specific effects as well. Finally, the field is also attempting transancestry GWAS in an attempt to increase prediction power across groups (Wojcik et al. 2019; Koyama et al. 2020). It is a pressing and active research problem to marcate the best solutions forward for the field, whether it be using transancestry GWAS meta-analyses to feed into PGS models which may suffer from the population stratification effects detailed above, or performing single ancestry GWAS and combining them in an approach such as PRS-CSx (Ruan et al. 2021) or creating methods that assume largely ancestry-specific effect sizes (Bitarello and Mathieson 2020; Marnetto et al. 2020). Our comprehensive review of the most state-of-the-art phenotypic prediction approaches indicates that methods assuming largely population-specific effect sizes show a marginal prediction improvement compared with methods that assume shared effect sizes between populations. The epistemological implication of these results is that there is a largely shared biology among humans with room for new mutations, gene-by-environment interactions, and selection events that can lead to some population-specific effects.

## Factors Affecting the Spatial Patterns of Genetic Variants

GWAS are used to identify trait-associated alleles that are in LD with causal alleles. Those causal alleles, which change the value of a complex trait, define the genetic component of a complex trait. Understanding the evolution of those causal variants around the globe is of fundamental importance to understand the spatial distribution of complex traits. The global spatial distribution of these causal alleles across the globe, as of other genetic variants, is broadly determined by degrees of genetic differentiation that are correlated with geographical distances (Ramachandran et al. 2005) and geographic barriers such as oceans, deserts, or mountains (Peter et al. 2020; Rosenberg et al. 2005). However, these patterns are not universal because population replacements and long distance admixture events (Peter et al. 2020) can break the relationship between geography and genetics. The joint impact of past demographic processes, natural selection, and environmental variation shape the geographical allele frequency distribution. Therefore, a good understanding of the evolutionary processes shaping spatial allele frequency variation is key to comprehend the distribution of complex traits in space. Here, we will discuss how past natural selection, population size changes, range expansions, nonrandom mating, archaic introgression, admixture, and the environment can change the frequency distribution of causal alleles.

The environmental conditions present in different regions can lead to selection of particular phenotypic trait values and lead to variation in complex traits across geographies that mirrors genetic variation. The relationship between the reproductive success (defined as fitness) of an individual and their trait value determines the type of selection acting on that trait. In humans, the reproductive success is a reflection of natural and biological factors along with cultural factors that also play a strong role. Conceptually, natural selection acting on a trait has been broadly classified as directional selection, stabilizing selection, and disruptive selection (Kimura 1983).

**Fig. 5:**
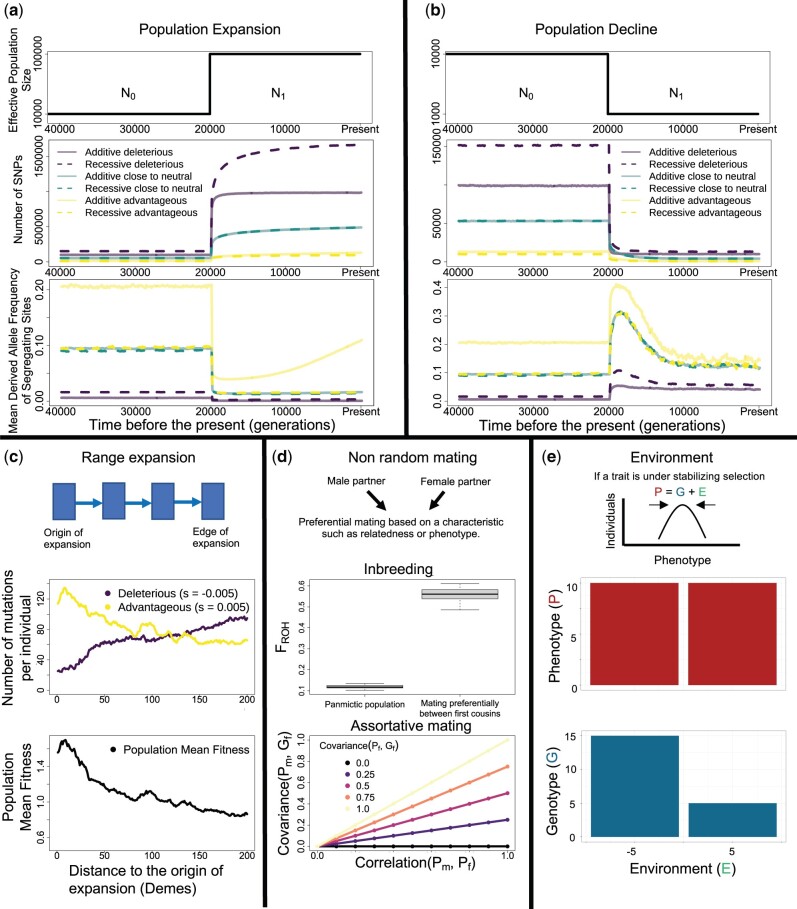
Impact of five different evolutionary processes on patterns of genetic variation. (*a* and *b*) Impact of a population expansion and a population decline on the number of segregating sites and the mean derived allele frequency of segregating sites across time. We computed those two statistics in simulations under the Poisson Random Field model under the two demographic models shown in the upper panel (Ortega-Del Vecchyo et al. 2016). The number of new independent mutations added each generation under this model follows a Poisson distribution with mean 2 N_0_u = 12,500 which changes to a Poisson distribution with mean 2 N_1_u after a population size change from N_0_ to N_1_ individuals that takes place 20,000 generations ago. The evolution of each independent mutation follows a Wright–Fisher model with selection where the selection coefficient assigned to each mutation is sampled from the distribution of fitness effects function at mutation–selection balance proposed by (Lourenço et al. 2011) using parameters inferred in humans (Huber et al. 2017). The mutations are classified based on their selection coefficient N_0_s as deleterious (N_0_s < –1), neutral (–1 < N_0_s < 1), or advantageous (N_0_s > 1). Results are shown for *h* = 0.5 (additive) and *h* = 0 (recessive) variants. (*c*) Impact of a Range Expansion on the number of advantageous and deleterious mutations per individual. We used the simulation program ADMRE (Peischl et al. 2013), where starting from five demes, the individuals colonize territories to form new demes by expanding toward the right side of the origin of the expansion. Only one new territory can be colonized each generation, and we show the results after 1,000 generations of a range expansion in the 200 demes next to the origin of expansion. There are two selection coefficients representing advantageous and deleterious mutations with the same magnitude of selection that can be assigned to each new mutation. The mean population fitness, shown in the lower plot, is calculated by averaging the population fitness of each individual which is equal to $w=\prod_{i}^{}(1+s_{i})$. These simulations were performed using the same parameters in (Peischl et al. 2013), but with a proportion of new mutations that are deleterious equal to 0.867 based on the proportion of mutations with a negative selection coefficient in the distribution of fitness effects used in Panel A and defining a space with 1000 territories. (*d*) Impact of nonrandom mating. We show two results depicting the impact of two forms of nonrandom mating that can be present in a population: Consanguinity and assortative mating. Upper panel: To illustrate the impact of consanguinity, we calculate the proportion of the genome that is autozygous, which is equal to the proportion of the genome inside a run of homozygosity (F_ROH). We performed 100 simulation replicates using SLiM (Haller and Messer 2019) for the scenarios “Panmictic population” and “Mating preferentially between first cousins” with 500 females and 500 males in the population, a mutation rate of 1.2e-8, a recombination rate equal to 1.0e-8, and a simulated region of 100 Mb. All the individuals are sampled at the end of the simulation. Relatedness does not play a role in the mating patterns under the “Panmictic population” scenario, whereas individuals are 100,000 times more likely to choose a first cousin rather than any other individual on the “Mating preferentially between first cousins” scenario. We estimated the runs of homozygosity using parameters defined previously (Ceballos, Hazelhurst, et al. 2018) allowing three heterozygous SNPs per run of homozygosity. Lower panel: To show the impact of assortative mating, we show how the genotypic predictor of phenotype in a female can predict the phenotype of its male partner. We denote the phenotype of the male and female partners as Pm and Pf, respectively, and we also define the genotypic predictor of the phenotype for the male and female partners as Gm and Gf, respectively. Note how the covariance between Pm and Gf increases as a function of the level of assortative mating, shown as higher values of correlation (Pm, Pf), and the effectiveness of the genotypic predictor, shown by the covariance (Gf, Pf). The lines plotted use an analytical equation derived from (Robinson et al. 2017). (*e*) Impact of the environment. If a trait is under stabilizing selection, the genetic architecture and environment will compensate for each other to maintain the trait at an intermediate value (Harpak and Przeworski 2021). Varying environmental effects on the phenotype in different locales can shift the genetic architecture, leading to higher frequencies of causal alleles in one locale compared with another, even with the trait value being the same in both.

The action of natural selection acting on traits can change the allele frequencies of causal alleles depending on the effect that the causal allele exerts on multiple phenotypes. The changes in frequency due to the action of natural selection, referred to as efficacy of natural selection, are jointly determined by the effective population size *N* at each time and the differences in fitness between individuals that possess a particular genotype. Broadly, those changes in frequency are higher when *N* is larger. We illustrate the impact of population size changes on allele frequency changes using simulations (fig. 5*a*). A population expansion increases the mean number of mutations that enter the population each generation (2Nu) increasing the number of segregating sites after the expansion (fig. 5*a*). The increased number of new rare mutations after a population expansion leads to an average decrease in the mean allele frequency on segregating sites for advantageous, neutral, and deleterious alleles (fig. 5*a*). These results are consistent with empirical results showing a large number of rare variants due to a recent population expansion in humans (Tennessen et al. 2012). On the other hand, a population decline decreases the number of segregating sites due to the impact of genetic drift driving a quicker loss or fixation of alleles (fig. 5*b*). In smaller population sizes, the action of genetic drift becomes a more important evolutionary process than natural selection to determine allele frequency changes. This property has been exploited in studies to detect trait-associated deleterious alleles in isolated groups that went through a population decline in the recent past, because some of those alleles can be found at a higher frequency compared with groups that have maintained a higher population size (Mahtani et al. 1996; Ober et al. 1998; Zeggini 2014; Locke et al. 2019). The impact of more complex population demographic scenarios that reconstruct past human history, including bottlenecks and recent population growth, on levels of deleterious genetic variation has received an important amount of attention in the literature (Gazave et al. 2013; Lohmueller 2014; Simons et al. 2014; Balick et al. 2015; Uricchio et al. 2016) with some studies also taking into account the effect of the dominance coefficient on levels of genetic variation (Simons et al. 2014; Balick et al. 2015), and finding that the average frequency of deleterious variants can actually remain constant after population bottlenecks due to occurrence of both fixation and loss events. Broadly, these studies are of particular importance to design GWAS that capture trait-associated variants based on the impact of past population history and dominance coefficients on levels of genetic variation (Lohmueller 2014; Simons et al. 2014). They are also relevant in association tests that pool information from rare variants inside a locus to detect the association of different loci with a particular phenotype (Uricchio et al. 2016).

The colonization of new territories due to range expansions also impacts the distribution of alleles under natural selection. The models of range expansion assume that populations inhabit a territory designated as the “origin of the expansion,” and that the populations expand outwards from this particular territory into the “edge of the expansion” (fig. 5*c* shows an example with one dimension). Newly colonized territories in range expansions start with a small number of individuals before growing and, therefore, there is a stronger genetic drift acting on mutations in territories that were colonized more recently (Peischl et al. 2016). This leads to an accumulation of deleterious variants compared with advantageous variants in the recently inhabited territories simply because a higher proportion of new mutations tend to be deleterious and the strong genetic drift is more important than the impact of natural selection on the change in allele frequency. We illustrate this in fig. 5*c* and show how this leads to a decreased mean population fitness on the edge of the expansion. The phenomena of range expansion has been recently invoked to explain that the distribution of deleterious variants across multiple groups is concordant with a series of range expansions out of Africa (Henn et al. 2016). However, the resulting impact on mutation load (and fitness) depends on the model of dominance, and under an additive effects model, very small differences in load are observed across groups (Simons et al. 2014; Henn et al. 2016).

Nonrandom mating is another process that shapes the distribution of causal alleles. This process varies historically and geographically and, therefore, impacts spatial allele frequency differences. Two forms of nonrandom mating that are important in human populations are consanguinity and assortative mating. Approximately 10% of the marriages across the globe involve couples that are related as second cousins or closer and their distribution varies across the globe (Bittles and Black 2010). The frequency of consanguineous marriages is shaped by both cultural practices and small population sizes (Ceballos, Joshi, et al. 2018). Such matings decrease the values of heterozygosity and increase the proportion of the genome inside a run of homozygosity (FROH), as illustrated with an example in figure 5*d*. Assortative mating is another important process that shapes the distribution of causal alleles. In line with this proposition, a recent study found that the trait-associated alleles for height, amongst other phenotypes, present in one individual are not only predictive of the phenotype in that particular individual but are also predictive of the phenotype of its mate when assortative mating is present (Robinson et al. 2017).

Mating with other hominids can also vary geographically and historically leading to varying degrees of archaic introgression introducing causal alleles into human populations. Some of the introduced alleles from Neanderthals have been linked to particular phenotypes such as skin lesions, neurological disorders, and sleeping patterns (Simonti et al. 2016; Dannemann and Kelso 2017). On the other hand, the impact of admixture between human populations is another area of recent interest. However, the particular effects from introduced causal alleles due to admixture are complicated to disentangle from the contribution of different environments, and from genotype by environment interactions.

The environment can also influence the spatial allele distribution due to correlations with allele frequencies in particular loci. Some of these correlations are due to the impact of natural selection where a particular environmental condition causes an increased selective pressure. This leads to events of local adaptation where individuals adapt to environmental conditions present in particular regions. Care must be taken when finding a correlation between an environmental variable and the frequency of an allele because this correlation can also be driven by neutral geographical processes such as an IBD or a recent expansion (Novembre and Di Rienzo 2009).

The interaction of the environment with natural selection can also affect the frequency of causal alleles. If the trait is under stabilizing selection or selection against the extremes of the trait (see section Selection on a Complex Trait), genetic architecture and the environment will compensate each other to maintain the trait at an intermediate value (Harpak and Przeworski 2021). As shown in figure 5*e*, if the environment decreases the trait value in one locale, then the causal alleles would increase in frequency to compensate. Similarly, if the environment increases the trait value in a different locale, then the causal alleles have a lower frequency to maintain the same average trait value in both locales. Note that this scenario does not require any gene by environment interactions, which on their own can also increase or decrease the frequency of a causal allele depending on the environment present in a certain locale.

## Factors Affecting the Spatial Patterns of Complex Traits

### Genetic, Biogeographical, and Sociocultural Axes

Similar to genetic variants, complex traits also present their own spatial patterns. Statistical and population genetics research has been concerned with identifying the genetic factors that may underlie such variation in trait values or disease incidence. How well do spatial genetic patterns mirror spatial trait patterns? What other correlated environmental patterns may be mirroring trait patterns? Complex traits can vary across genetic and environmental axes. Simply conceived, the environmental axis can be biogeographical or sociocultural. Biogeographical axes are structured by the conditions along which climatic and ecological variables may change (latitude, longitude, altitude, and habitat). Sociocultural axes are arranged by differences in diet, income, access to education, access to healthcare, ethnicity, and experience of racism and discrimination. The genetic axes capture variation in allelic frequencies and in levels of genomic homozygosity. This genetic variation at a given time is shaped by ancestral demographic histories and is often captured using the concept of genetic ancestry. Biogeographical, sociocultural, and genetic axes are often correlated in complex ways, depending on the specific environmental and cultural history of a place and people. Therefore, disentangling them and their interactions presents a very difficult problem. In particular, gene–environmental correlations (e.g. between a specific ancestry and discrimination) can cause the environment to lead to trait variation that can then be mistakenly perceived as caused by genetics (Harpak and Edge 2021).

### A Look at the Case of Hypertension

The case of hypertension will provide an illustrative example. Hypertension is observed at a higher rate in the United States in African Americans compared with European Americans (Cooper and Rotimi 1997). One may hypothesize that African ancestry presents a genetic predisposition to developing hypertension. However, African ancestry is also correlated with racial categories in the United States that have meant different opportunities and access to health care for black and white people which may cause differences in hypertension as well. These two conjectures raise the question: Are the hypertension patterns observed in African Americans and European Americans caused by differences in their genetic predisposition to hypertension or are they caused by sociocultural differences that restrict opportunities and access to health care for African Americans?

**Fig. 6.**
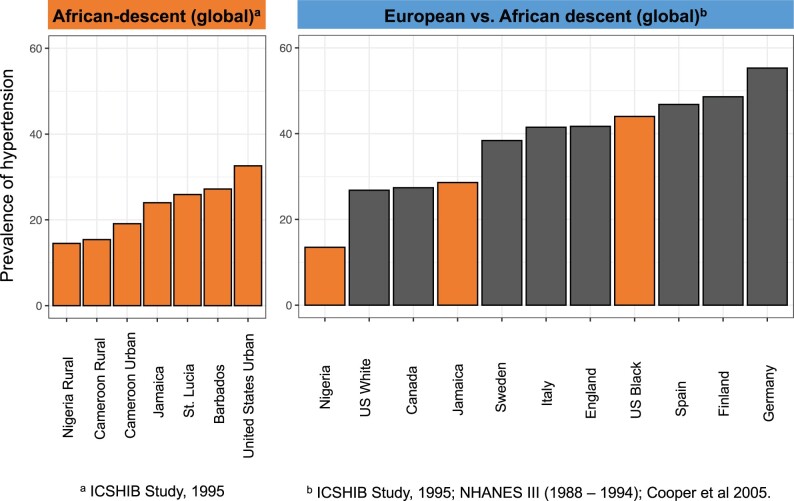
Prevalence of hypertension worldwide. Age and sex-adjusted prevalence of hypertension is shown. Individuals of African descent were drawn from The International Collaborative Study of Hypertension in Blacks (ICSHIB, 1995) and the National Health and Nutrition Examination Survey III (NHANES III, 1988–1994) (Cooper et al. 1997; Wolf-Maier et al. 2003). For other individuals from North America and Europe, eight surveys were previously combined by Cooper et al. (2005). Collectively, the studies enrolled over 85,000 participants and individual studies ranged from 1,800 to 23,000 participants. Hypertension is defined as having high blood pressure (140/90 mmHg or taking antihypertensive medications). Bars are colored by African (orange) and European (gray) descent.

Slavery, colonization, and decolonization along skin color lines are processes that have underscored global history in the last 500 years. To understand traits like hypertension, we have to understand how those dynamics impacted the individuals studied. One way to do so is using a comparative method to look across populations with different histories (e.g. African Americans vs Africans in Africa). Indeed, as a wider selection of people have been sampled, it has become evident that Africans in Africa, especially rural Africans, actually have some of the lowest levels of hypertension in the world (Cooper et al. 1997, 1999; Guwatudde et al. 2015) (fig. 6). Hypertension rates are led in the world by Finland and Germany, followed by other European countries, and then by the United States and Canada (Wolf-Maier et al. 2003; Cooper et al. 2005). These studies of the disparities in hypertension rates for individuals of African descent living in Africa versus living in the United States highlight the importance of considering changes in environmental factors and the interactions between genetics, physiology, and the environment in causing chronic diseases and more broadly shifts in complex traits values. They certainly point against a simple one-to-one causal mapping between African ancestry and a complex trait such as hypertension.

Another study highlighting the importance of sociocultural axes found that blood pressure in Puerto Rico showed an association with skin color as defined through social classification using the ethnographic method of cultural consensus analysis which estimates how respondents are perceived by others in social interactions but not skin color as measured through reflectance spectrophotometry (Gravlee et al. 2005). The authors conclude that social classification, but not skin pigmentation, is associated with systolic and diastolic blood pressure through a statistical interaction with SES. In this case, although hypertension patterns mirror genetic patterns as well as social classification patterns, the causal relationship appears to be between social discrimination and hypertension.

Several present-day societies have been structured along lines of color and race with respect to social standing and opportunity which can lead to observable variation in complex traits. Within the general phenomenon of cultural contact, diets have also gone through larger turnovers for certain people and places during colonization or due to westernization driven partly by imperialism. This look at the spatial patterns of hypertension and blood pressure in a global context illustrates that purely genetic explanations for complex trait variation should be avoided when other correlated sociocultural variables, or an interaction between genetic and sociocultural variables, may be more likely to explain the patterns of variation within a single country with a specific history where discrimination has been correlated with skin color (a genetically influenced trait) or where big environmental shifts such as those in diet have taken place for certain groups.

### Genetics and Environment Shape Complex Trait Distributions

The joint action of genetics and the environment determine the final trait value in a given individual or group. Due to this, it is important to consider the role of the environment when performing phenotypic predictions based on the genetic information from PGS. To illustrate this point, we will study the relationship between PGS and trait values in groups distributed across space. First, we will consider two populations in Japan studied with respect to their trait distributions and their PGS distributions. A recent study looked at a number of different complex traits in individuals from mainland Japan and nonmainland Japan (Sakaue et al. 2020). The authors randomly split the Biobank of Japan into a discovery group for the GWAS and a validation group for the polygenic prediction, with both groups having equal numbers of individuals from the mainland and nonmainland clusters. To account for population stratification, they included sex, age, 20 principal components, and whether an individual was included in the mainland cluster or the nonmainland cluster as covariates in the GWAS. They found that the difference in average height among these two populations (mainland and nonmainland) was in the same direction as the difference in the average PGS for height among them (higher in the mainland). On the other hand, although the average BMI is higher in nonmainland Japan, the average PGS for BMI is lower in nonmainland Japan compared with the mainland. Overall, they found no correlation between the PGS deviation and phenotypic deviation across 45 quantitative and 25 binary (diseases, smoking/drink habits) traits tested. Longitudinal data revealed that BMI in Okinawa (most of the nonmainland) only began to exceed that of the mainland starting less than a decade ago. The authors speculate that the phenotype in this case may be affected by rapid environmental changes such as dietary changes (i.e. Westernization) after World War II, which Okinawa experienced.

For two populations in Colombia, Antioquia (Mestizo) and Choco (Afro-Columbian), Chande et al. (2020) found an overall concordance across several traits (WHI, height, BMI, hair color, eye color, inflammatory bowel disease, ischemic stroke, mortality in heart failure, immunity to malaria) between PGS predictions and observed anthropometric and epidemiological profiles. The authors used SNP trait associations from the NHGRI-EBI GWAS catalog which includes all GWAS associations across a large number of traits and study populations. However, when studying 12 high impact diseases, they found some notable exceptions. T2D shows the largest difference between PGS prediction and observed disease prevalence. Choco has a higher average PGS (or predicted genetic risk) but lower prevalence compared with Antioquia. Chronic kidney disease shows a similar pattern, but not as extreme. The authors point to protective environmental factors, with respect to diet and lifestyle, mitigating the risk of T2D and chronic kidney disease in Choco. Susceptibility to malaria showed a similarly large difference between PGS (predicted risk) and observed prevalence caused by both *Plasmodium vivax* and *Plasmodiumfalciparum*. Predicted genetic risk for malaria infections is lower in Choco, whereas the malaria prevalence is higher in Choco compared with Antioquia because both *P. vivax* and *P. falciparum* are more prevalent in Choco. Overall, these results point to the importance of environmental factors such as diet, lifestyle, and the abundance of pathogens as elements that can play a role in causing discrepancies between polygenic phenotypic predictions and trait values.

Next, we will consider how a widely studied trait, educational attainment (EA), and its PGS varies by birth year in the UK Biobank. Abdellaoui et al. used summary statistics from a meta-analysis performed on a large number of cohorts of European descent ancestry to compute PGS for EA. Principal components computed on their prediction cohort (UK Biobank) were later regressed out of the PGSs to correct for stratification effects. Across the country, EA in Great Britain shows a positive correlation with birth year, whereas the PGS for EA shows a negative correlation with birth year (Abdellaoui et al. 2019). That is, although younger individuals have a lower PGS for EA, they nevertheless have a higher EA. This observation points to the potential importance of environmental factors in values of EA, as the trait is higher in younger individuals despite the PGS being lower.

Given the detailed prior discussion of problems of biased estimation of genetic effects and of variable powers of prediction, the reader is justified in wondering if the results presented in the section may be due to the same. This is certainly possible and remains an area of active research. Nevertheless, we think that these examples are instructive to make the point that a trait’s value results from a combination of genetic and environmental processes. An often underlooked cause for the discrepancy between a PGS prediction and an actual trait value is the role of environmental factors. It is in this sense that a PGS can never be considered determinative of a trait value, as it is only one aspect of trait/disease manifestation (Levins and Lewontin 1985). As such, spatial genetic structure does not always mirror spatial trait structure, as the relationship between the two is ultimately determined by the underlying environmental structure.

### Selection on a Complex Trait

As described before, the spatial environmental structure is an important factor to take into account when describing the evolution of complex traits. The environment can change the distribution of a trait in a particular location due to the effect of natural selection. In this section, we will discuss the main findings of studies conceptualizing and elucidating the roles of stabilizing, disruptive, and directional natural selection acting on complex traits.

### Stabilizing and Disruptive Selection

The contributions of stabilizing and disruptive selection have been recently studied in a set of complex traits using data from the UK Biobank (Sanjak et al. 2018). Sanjak et al. (2018) applied the framework developed by Lande and Arnold (1983) to quantify if stabilizing selection and disruptive selection are acting on a set of traits, including height and BMI. The main idea of this framework is to perform a linear regression of fitness onto a particular phenotypic value and its squared phenotypic value. The values of the quadratic term’s coefficient, which estimates the effect of the squared phenotypic value in the regression, can be informative of the impact of stabilizing and disruptive selection (Lande and Arnold 1983). Stronger stabilizing selection and disruptive selection cause larger negative and positive squared regression coefficients, respectively. Sanjak et al. (2018) use the relative lifetime reproductive success (rLRS), which is the individual lifetime reproductive success divided by the mean value in a particular cohort, as a proxy for fitness to perform a series of sex-specific linear regressions for a set of complex traits on rLRS. The authors find evidence of stabilizing selection based on the negative squared regression coefficient obtained in 47 of the 64 regressions performed. These included traits such as height, weight, bone mineral density, waist circumference, and basal metabolic rate. Sanjak et al. (2018) find a positive squared regression coefficient in 5 out of the 64 regressions pointing to a limited presence of disruptive selection in the traits analyzed. Broadly, these analyses suggest a widespread action of stabilizing selection acting on human complex traits based on the UK Biobank data. Changes in the strength of stabilizing and disruptive selection in different regions across the world could be a factor driving spatial phenotypic changes. Further studies could help to clarify the role of those two modes of selection on different traits around the globe.

### Directional Selection

Populations can adapt to new environmental conditions through directional selection on complex traits. This evolutionary force has been hypothesized to drive phenotypic differences between different populations and, therefore, has motivated a vigorous line of research during the past decade. However, the detection of polygenic adaptation is challenging because the signal depends on frequency shifts at many alleles. There will not be a LD signal around the alleles that is indicative of the action of natural selection if those allele frequency changes are small. This particular issue has been investigated using analytical theory (Höllinger et al. 2019) and simulations (Thornton 2019) in models where there is a shift in the optimum value of a trait. The results from (Höllinger et al. 2019) show that the background mutation rate (4Nu) determines the dynamics of adaptation, where u is the sum of the mutation rate across all causal loci. When the background mutation rate is smaller than 0.1, then adaptation takes place by changes in frequency from low to high frequency in a single allele. As 4Nu increases, the dynamics of adaptation is based on subtle allele frequency changes across a larger set of loci. Following this finding, Kevin Thornton (2019) performed simulations that included a shift in the optimum value of a trait. He found that it is harder to detect signals of adaptation using patterns of LD when the background mutation rate is large, which is 4Nu = 100 in this case.

Results from GWAS have allowed the development of new statistical tests to find evidence of directional selection by incorporating information from trait-associated alleles and their effect sizes. Broadly, these tests use information from coordinated allele frequency shifts that are common when there is a large background mutation rate. One of the pioneer studies to incorporate GWAS information to detect directional selection found that height increasing trait-associated alleles have a significantly higher frequency in Northern Europeans compared with Southern Europeans (Turchin et al. 2012). Further studies have incorporated effect sizes of trait associated SNPs to test for the evidence of directional selection. Berg and Coop (2014) analyzed the overdispersion of genetic values (e.g. an excess of variance in the genetic values among populations) due to natural selection driving changes in the trait values in a particular population. Similar approaches have also been used to test for the evidence of directional natural selection in admixed populations. In particular, Racimo et al. (2018) developed Polygraph to infer the impact of natural selection in a previously defined admixture graph, which represents the history of divergence and admixture events among different populations as a set of branches. The length of each branch is determined by the amount of genetic drift that has taken place, and Polygraph tests for the evidence of natural selection in particular branches by testing for large deviations in the mean genetic values in particular branches of the graph given the amount of drift in those branches of the tree (Racimo et al. 2018). Finally, the trait-singleton density score statistic has been developed to detect signatures of polygenic adaptation by using the distances to the first singleton from positions in the genome that have causal alleles (Field et al. 2016). The intuition behind this statistic is that recent natural selection reduces the time to a first coalescent event between individuals that carry an advantageous allele. This reduction decreases the number of singleton mutations next to the advantageous allele which is reflected in an increased distance from the advantageous allele to the first singleton mutation. If this distance is significantly higher on average taking many causal alleles across the genome that change the trait value in a particular direction, then we can interpret this pattern as a sign of polygenic adaptation acting on a trait (Field et al. 2016).

Despite the statistical soundness of tests to detect directional selection events, there have been major concerns regarding the validity of the current approaches available to detect signatures of directional natural selection due to population stratification, the transferability of effect sizes between populations, and ascertainment biases on the genotyping chip (Martin et al. 2017; Novembre and Barton 2018; Berg et al. 2019; Sohail et al. 2019). Additionally, the environment can drive changes in the trait values that do not reflect changes in the genetic values, as seen in the section “Genetics and Environment Shape Complex Trait Distributions.” Moreover, under stabilizing selection, shifting environmental effects can drive genetic value changes without the action of directional natural selection changing phenotypic values (Harpak and Przeworski 2021).

## Conclusions and Future Directions

### The Path Forward—Lessons from Studies on Human Height

A clear understanding of the factors that lead to phenotypic differences requires a careful assessment of how sociocultural, biogeographical, and genetic factors drive those changes. As an example, we can think of the impact that different variables associated with these factors have on human height. SES has a direct impact on net nutrition, the most important nongenetic factor affecting height (Perkins et al. 2016). Exposure to infectious diseases, such as intestinal parasites, is an example of a biogeographical factor that has an effect on height (Perkins et al. 2016). Our current best approach to interpret the factors jointly contributing to the evolution of height differences across the globe requires a careful analysis of the socioeconomic, biogeographical, and genetic factors in different groups of individuals living in different environments. This strategy has been taken in recent years and has helped us to identify population-specific and globally common genetic variants that influence height (Wood et al. 2014; Zoledziewska et al. 2015; Akiyama et al. 2019; Asgari et al. 2020) and gain insights into the signatures of natural selection acting on human height.

GWAS of height have been performed on cohorts of individuals from single countries such as United Kingdom (Churchhouse 2017), Japan (Akiyama et al. 2019), Peru (Asgari et al. 2020), and Sardinia (Zoledziewska et al. 2015), as well as in mega-cohorts meta-analyzing several smaller cohorts (Wood et al. 2014). The studies focused on single populations from one country have been fundamental to identify genetic variants with a large effect on height values that are very rare or not found even in large-scale studies on different populations. The studies on Sardinia have identified a rare variant that reduces height by 4.2 cm and has a frequency smaller than 0.01% in other populations (Zoledziewska et al. 2015), whereas the study on Peruvian populations has identified another rare variant that reduces height by 2.2 cm (Asgari et al. 2020). Indeed, a recent study has demonstrated that the vast majority of loci that differ in frequency between traditional continental groups are rare in one group and virtually absent from others (Biddanda et al. 2020). These population-specific variants would hinder our height phenotypic prediction if GWAS summary statistics from another population are used to perform the predictions, simply because these variants are either absent or have a very low frequency in other populations and would not be captured in their GWAS. The expansion of GWAS on height to more populations will probably reveal more population-specific variants impacting height that will ultimately allow us to understand the genetic architecture of height in each studied population and globally.

Directional selection on complex traits has been hypothesized to be an important factor driving phenotypic differences in height among and between populations. There have been suggestions that the height differences between Northern and Southern European populations are driven by polygenic adaptation. This problem was first approached using GWAS summary statistics from the GIANT consortium, a meta-analysis containing samples from European populations (Turchin et al. 2012), where the authors found that alleles associated with an increased effect on height are at higher frequencies in Northern Europeans compared with Southern Europeans. Two studies independently found that population stratification in GIANT, which artificially increases the effect size of variants differentiated along the same environmental gradient driving phenotypic differences, generates false signals of polygenic adaptation (Berg et al. 2019; Sohail et al. 2019). This problem has been tackled using effect size data from a GWAS study performed in a distant population from the BioBank Japan, which is not affected by the population stratification present in the studied populations, and has found signals of polygenic adaptation for smaller stature in the ancestors of modern Sardinians (Chen et al. 2020). This solution is not optimal because the phenotypic predictions are less accurate when using a panel from a distant population and leads to a reduced power to detect events of polygenic adaptation. Another key factor in these analyses is to use a large panel with homogeneous ancestries to avoid false signals of polygenic adaptation (Refoyo-Martínez et al. 2021).

### A Historical Lens to Guide the Future

A look to the past can help contextualize the research trajectory so far, and provide a guide for research in the future. Populations as used in evolutionary genetics today can be directly linked to a racialized worldview that was inherited by the field of genetics from earlier academic and public discourse alike (Yudell 2014; Saini 2019). During the modern synthesis, Dobzhansky claimed that “races can be defined as populations which differ in the frequencies of some gene or genes,” trying to define race within a population rather than the older typological framework (Dunn and Dobzhansky 1946). This is when race went from being an object of study in the pre-WWII period to becoming more subtly embedded in the population-focused methodology of the science post-WWII (El-Haj 2007; Yudell 2014). It is in that sense, and with this history in mind, that racial thinking pervades our science and our lived experience today (Brattain 2007; Fullwiley 2014). A worldview was first created to justify the slavery of Africans and the colonization of large parts of the world based on color, and cemented in intellectual frameworks in natural philosophy, biology, and anthropology. Then came genetics, both inheriting this legacy and playing a role in pushing it forward through the joint development of eugenics, genetics, and other forms of racial sciences (Yudell 2014; Saini 2019). The earlier research (pre-WWII) worked under assumptions of large innate differences between human races along a hierarchy reflecting the inequities visible under color-based segregation and colonial rule, and geared toward understanding differences between, for example, “negroes” and “whites,” and the betterment of the “superior” race through elimination of contaminants from other races (Yudell 2014; Saini 2019).

History shows us that science and scientists can be operating under extrascientific forces, and as such, the process of science has a fundamentally subjective aspect to it. This is a lesson for us today. As we familiarize ourselves with this history, we can understand the role it played in motivating and being generated by the development of concepts and ideas in genetics, statistics, and related disciplines. This will encourage us to revisit assumptions or ambiguities regarding the existence of populations (Panofsky and Bliss 2017), and the interplay between genes and their context (Gravlee et al. 2009). In the short-term, this implies: 1) consciously asking why and how to group individuals for any given research question, communicating the reasoning behind these groupings in a research manuscript, and being flexible to different and new ways of grouping/ungrouping informed by results, 2) simultaneously modeling genetic and environmental variables, seeking environmental variables in data sets and measuring them in new field work, 3) forcing ourselves to be explicit about model assumptions and communicating these early in a manuscript, 4) doing necessary work in writing and divulgation to reduce oversimplification of results in the public eye and the chances of work being taken up by racist and dangerous narratives, 5) being value-neutral about traits and diseases and incorporating input from patient communities, 6) avoiding highly deterministic modes of thinking and writing, for example, with respect to mapping between a PGS and trait/disease manifestation. In the long-term, future research should focus on models that do not artificially separate genetics and the environment and work toward an updated concept of heritability that does the same. Furthermore, steps should be taken toward a genetics education and research model that strives to be more transdisciplinary to aid in increased awareness and skills among geneticists to tackle these issues. Along the same lines, we echo the call for “standards for research design that acknowledge the historical, political, and social context of phenotypes under study” (Richardson et al. 2019).

Although biology plays a role in shaping complex trait variation, it should not be assumed that it follows some natural hierarchy across all traits along color lines and can, therefore, be used to justify discrimination and racism. Indeed, methods that assume population-specific genetic effects and biology show a marginal improvement to predict complex traits and diseases across human diversity over methods that assume largely shared genetic effects. These results argue in favor of largely shared biological functions among individuals living in different geographies, with room for population-specific effects and interactions with the environment. As such, complex trait studies that include individuals living in different regions and environments will be crucial to get an accurate picture of the factors shaping complex trait diversity across the globe, helping identify shared and private aspects of genetic architecture and interactions with the environment. Such diverse sampling and methodological and conceptual advances will take us further in understanding genetics as mediated by the biogeographical and sociocultural environment to generate the mosaic picture of complex trait variation we see today.
